# Takayasu Arteritis Complicated by Ischemic Colitis: A Case Report

**DOI:** 10.3400/avd.cr.21-00129

**Published:** 2022-03-25

**Authors:** Shigeyuki Yamashita, Kanetsugu Nagao, Toshio Doi, Shigeki Yokoyama, Akio Yamashita, Kazuaki Fukahara, Naoki Yoshimura

**Affiliations:** 1 First Department of Surgery, University of Toyama, Toyama, Toyama, Japan

**Keywords:** Takayasu arteritis, ischemic colitis, vascular surgery

## Abstract

Takayasu arteritis is an inflammatory disease of the aorta and its major branches, which results in stenosis and aneurysm formation. Lesions of the abdominal aorta and renal arteries are common. Nevertheless, lesions of the celiac and superior mesenteric arteries are less common. Since the inferior mesenteric artery is usually preserved and functions as a collateral pathway, developing intestinal ischemia is very rare in patients with Takayasu arteritis. In this study, we report the case of a patient with Takayasu arteritis complicated by ischemic colitis. The patient was treated with surgical repair, which resolved the patient’s symptoms.

## Introduction

Takayasu arteritis is an inflammatory disease of the aorta and its major branches, which result in stenosis and aneurysm development. Given that the site of inflammation varies from case to case, the clinical manifestations of Takayasu arteritis are diverse. In Japan, the ascending aorta to the arch and its branches are usually affected. Moreover, head and neck symptoms such as dizziness or upper extremity symptoms such as pulselessness are common.^1,2)^ Lesions of the abdominal aorta and renal arteries are also common. However, lesions of the celiac artery (CA) and superior mesenteric artery (SMA) are less common. In the present case, we surgically treated a patient with Takayasu arteritis complicated by ischemic colitis due to the stenosis of the abdominal aorta and CA and occlusion of the SMA.

## Case Report

**Figure 1:**
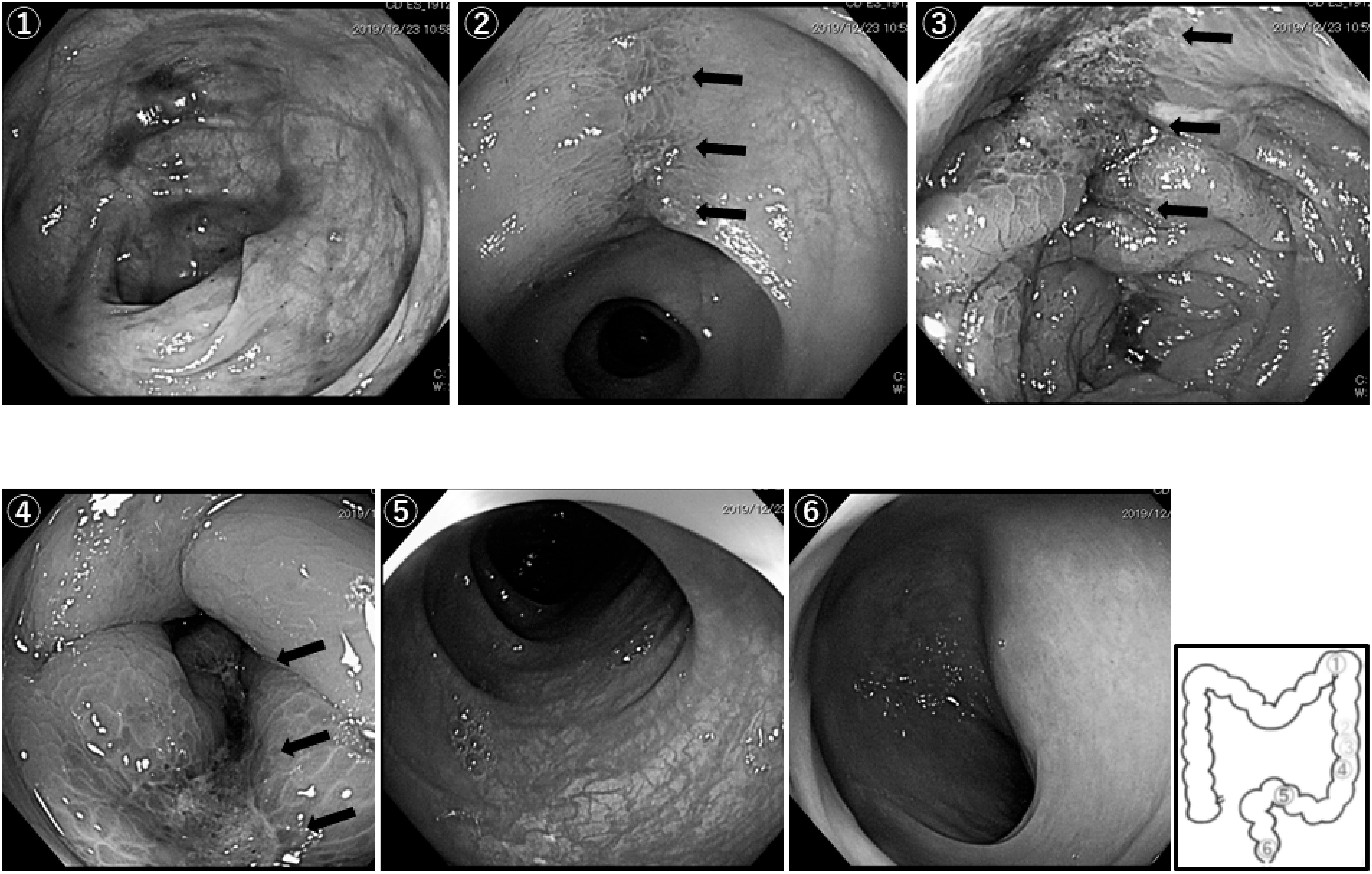
Fig. 1 Colonoscopic findings show no lesions in the left colic flexure, sigmoid colon, or rectum. However, longitudinal ulcers (arrows) in the descending colon are shown.

**Figure 2:**
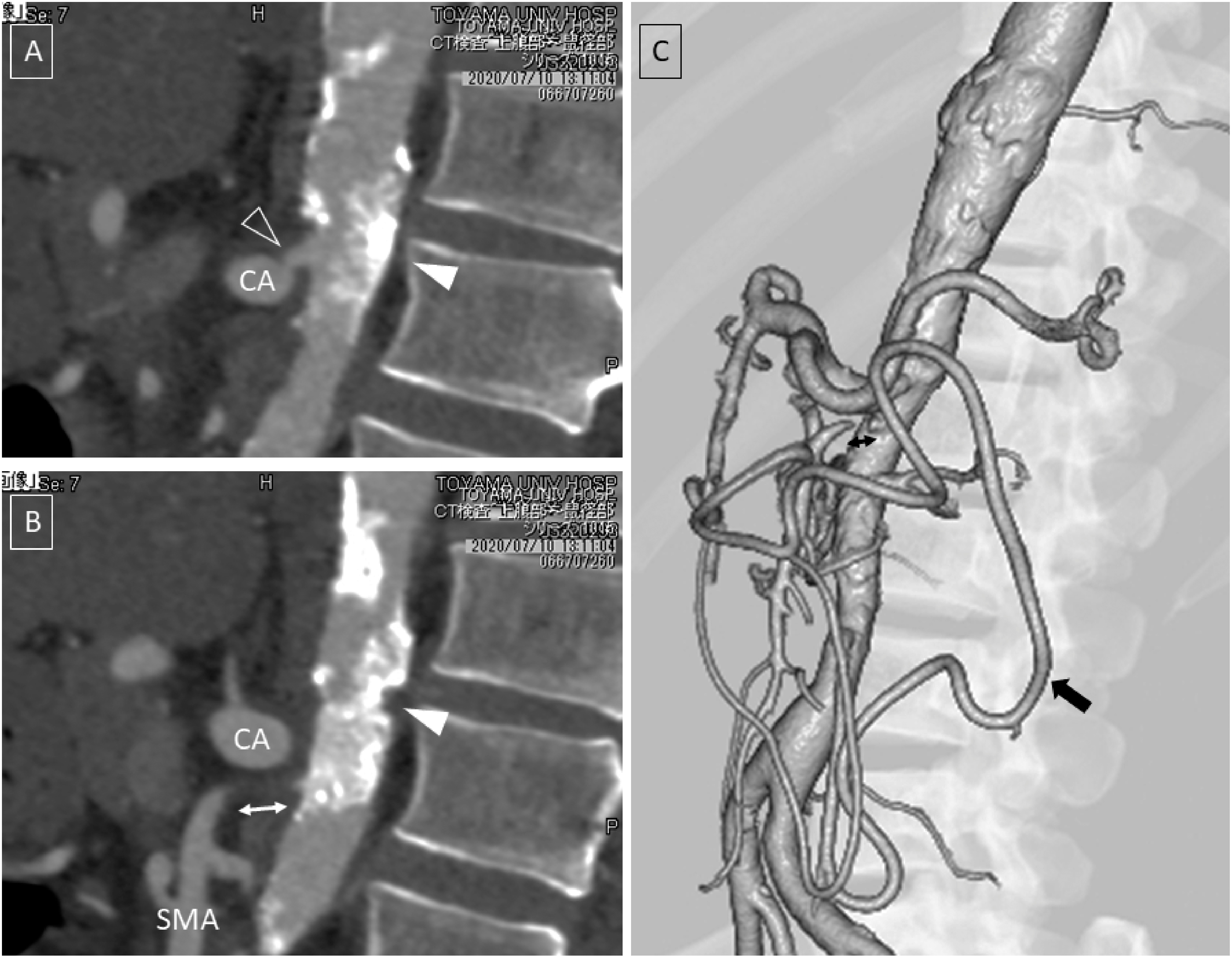
Fig. 2 Preoperative enhanced computed tomography (**A** and **B**, a sagittal view; **C**, a volume-rendered image). (**A**) Calcified stenosis in the abdominal aorta (filled arrowhead) and stenosis of origin in the celiac artery (unfilled arrowhead). (**B**) Calcified stenosis in the abdominal aorta (filled arrowhead) and occlusion in the superior mesenteric artery (SMA) (double-headed arrow). (**C**) Collateral blood vessels from the developed inferior mesenteric artery to the SMA can be seen (arrow), and the SMA is occluded (double-headed arrow).

The patient was a 43-year-old woman. At the age of 16 years, she was diagnosed with Takayasu arteritis due to fever of unknown origin and carotid wall thickening and had been taking prednisolone for the next several years. Upon resolution of her symptoms, this medication was discontinued. At the age of 40 years, a routine checkup revealed that she had persistently mildly elevated C-reactive protein (CRP) (0.2–0.4 mg/dL) levels and worsening hypertension. Furthermore, a follow-up computed tomography (CT) revealed calcified stenosis of the abdominal aorta and stenosis of the CA and SMA. She was diagnosed with recurrent Takayasu arteritis and administered prednisolone 15 mg/day and methotrexate 12 mg/week. Aspirin 100 mg/day was administered for CA and SMA stenosis. She continued these medications until the age of 43 years when she experienced a sudden onset of left-sided abdominal pain and bloody stool and visited the internal medicine department of another hospital. Blood test results revealed normal CRP and erythrocyte sedimentation rate levels (0.06 mg/dL and 6 mm/h, respectively) and a mildly elevated white cell count of 9,980/µL. Colonoscopy showed no rectal lesions. However, longitudinal ulcers were found in the descending colon and diagnosed as ischemic colitis (**Fig. 1**). After approximately 1 week of conservative treatment, the symptoms disappeared. However, 6 months later, she experienced a recurrence of left-sided abdominal pain and bloody stool (a small amount of fresh bright-red blood in stool) and visited our hospital. Upon physical examination, her blood pressure was 186/82 mmHg, and there was no difference in blood pressure between the right and left upper limbs. No vascular murmurs were heard over the neck. The abdomen was flat and soft, with mild tenderness on the left side of the abdomen. The bowel sounds were physiological, and vascular murmur was heard in the epigastrium. Bloody stools were not present at the time of admission. Palpation of the common femoral artery showed a weak pulse. Additionally, the ankle–brachial pressure index decreased when compared with normal levels (right, 0.56; left, 0.58). Contrast-enhanced CT showed worsening of the stenosis of the abdominal aorta and CA and occlusion of the SMA. The inferior mesenteric artery (IMA) was well-developed and formed a collateral route to the SMA (**Fig. 2**). Thus, we diagnosed the patient with ischemic colitis associated with stenosis of the abdominal aorta and CA and occlusion of the SMA. We determined that surgical revascularization was necessary.

The method of revascularization was discussed. Since the IMA was functioning well and the infrarenal abdominal aorta was not affected, there was a high possibility that only a bypass between the descending thoracic aorta and infrarenal abdominal aorta would improve intestinal ischemia. The patient was young, and the possibility of future involvement of the IMA could not be denied. Thus, we decided to add reconstruction of the CA and SMA to maintain sufficient and long-term intestinal blood flow. Regarding the proximal anastomosis site, there were no obvious lesions in the descending thoracic aorta. Additionally, there was a possibility of requiring coronary artery bypass surgery or aortic valve replacement in the future. Consequently, we decided to perform an interaortic bypass using an anatomical route with the descending thoracic aorta as the proximal anastomotic site.

**Figure 3:**
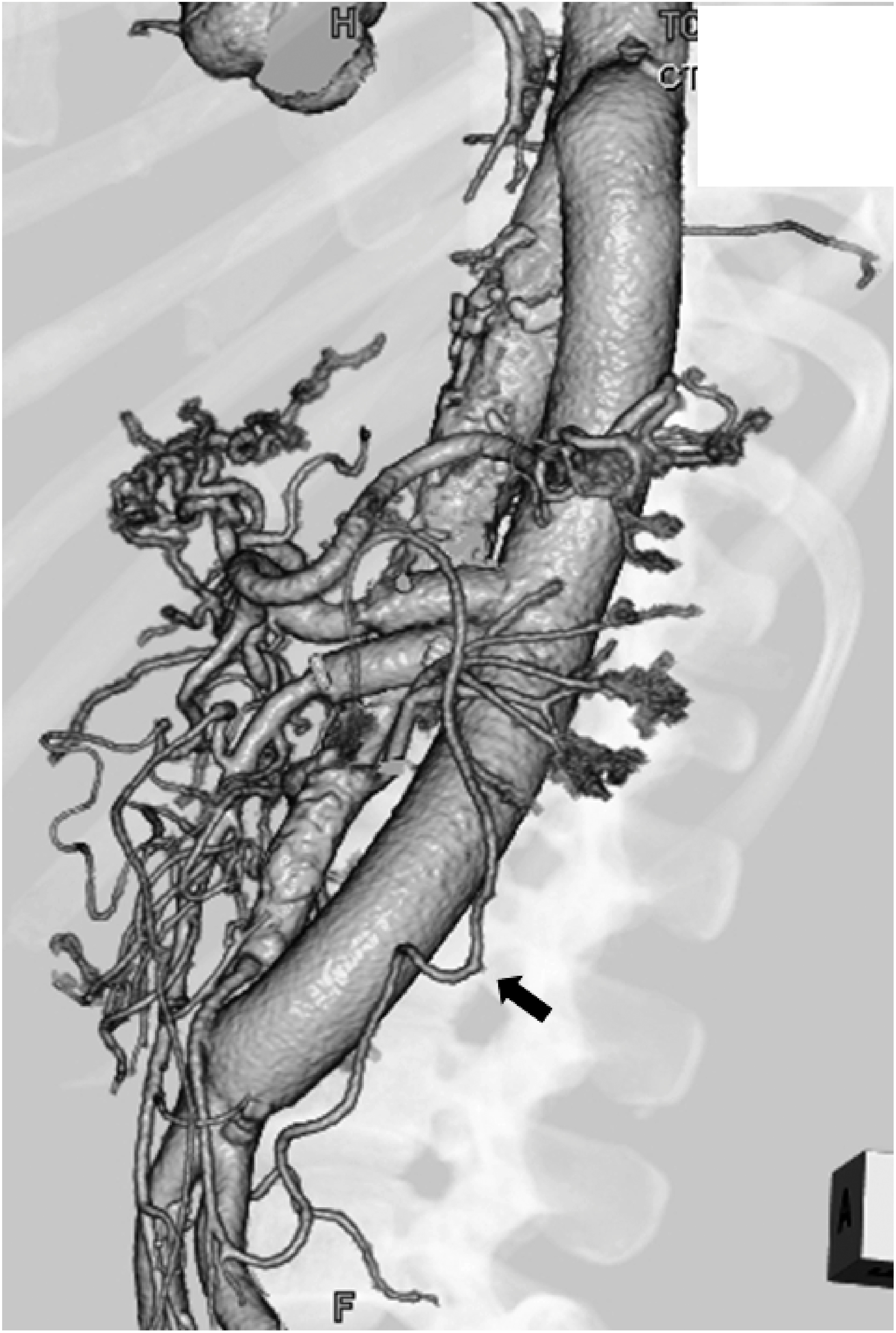
Fig. 3 Postoperative enhanced computed tomography (CT) (a volume-rendered image). No major anastomotic abnormality can be observed, and the celiac artery and superior mesenteric artery are patent. The collateral vessels from the inferior mesenteric artery seen in the preoperative CT are regressed (arrow).

The operation was performed through a Stoney’s incision (left thoracotomy at sixth intercostal space). The retroperitoneal space was approached through a pararectal incision. Descending thoracic aorta–abdominal aortic bypass and CA/SMA reconstruction were performed with partial extracorporeal circulation (via femoral arterial and femoral venous access and CA perfusion with another pump). On gross examination, both proximal and distal anastomotic sites showed mild atherosclerotic changes, with no obvious wall thickening or adhesion to the surrounding tissue. The anastomotic sites were reinforced with a bovine pericardium patch. The operative time was 357 min, and the intraoperative blood loss was 90 mL. Pathological findings of the aortic wall stained with Elastica van Gieson showed that the elastic tissue was torn off in the fibrotic area. Moreover, there were fibrous growth and mild lymphocyte and plasma cell infiltration in the adventitia and extravascular connective tissue. Postoperative CT showed no anastomotic pseudoaneurysm, and the CA and SMA were patent (**Fig. 3**). The patient was discharged on postoperative day 17, without any major complications. Although the patient had been treated with prednisolone, methotrexate, and aspirin preoperatively, stenosis of the CA and SMA worsened. Thus, besides these drugs, tocilizumab at a dose of 162 mg/week was added postoperatively. One year after surgery, the patient has progressed well without recurrence of abdominal symptoms.

## Discussion

Takayasu arteritis is a disease that causes vasculitis of the aorta and its first branch, coronary arteries, and pulmonary arteries. The subclavian artery is the most frequently affected artery (93%), followed by the aortic arch (65%) and carotid artery (58%). By contrast, its incidence in the abdomen is slightly lower: 47%–80.2% in the abdominal aorta; 38%–67.5% in the renal artery; and 4.4%–18% and 11%–21.4% in CA and SMA, respectively. Conversely, the IMA is less frequently involved (2%–6.3%).^2–5)^

Besides the systemic symptoms such as fever and malaise (74.9%), head and neck symptoms such as dizziness and headache (47.5%) and upper extremity symptoms (66.4%) are common in patients with Takayasu arteritis.^1)^ However, symptoms of intestinal ischemia are extremely rare. This rarity is thought to be because the IMA, which can serve as a collateral source, is often preserved even when the CA and SMA are involved.^3)^ In the present case, although there was stenosis and occlusion in the CA and SMA, no lesion in the IMA was found, which became a well-developed collateral source. Nevertheless, severe stenosis in the abdominal aorta at the level of the diaphragm is thought to have caused ischemic colitis.

There have been several case reports of patients with Takayasu arteritis accompanied by abdominal pain. However, the causes varied in these cases. Intestinal ischemic symptoms due to abdominal aortic branch involvement were the most frequently reported.^6–8)^ However, other symptoms comprise pain due to intraabdominal aneurysms (including pseudoaneurysms)^9)^ and pain due to active inflammation of the abdominal aorta, CA, and SMA. Inflammatory bowel disease (IBD) can also cause abdominal pain.^10)^ IBD in patients with Takayasu arteritis is often difficult to diagnose at the time of initial presentation because endoscopic findings are often nonspecific to IBD.^10)^ The endoscopic findings in the present case showed longitudinal ulcers in the descending colon, which is a characteristic of ischemic colitis. Moreover, there was no rectal lesion, which is often seen in IBD. The diagnosis of ischemic colitis was established in combination with the contrast-enhanced CT findings.

In patients with Takayasu arteritis, especially when ischemic colitis is present at the time of the first presentation, the rarity of the disease has led to delayed diagnosis and death in some cases. In one case, the rapid progression of intestinal ischemia resulted in extensive intestinal necrosis, and the patient died despite emergency surgery.^6)^ In another case, prolonged mesenteric angina resulted in significant weight loss, and the patient was treated intensively. Nevertheless, he did not recover sufficiently and died.^7,8)^ Similarly, in the present case, at the first onset of abdominal symptoms, the symptoms were transient and resolved with conservative treatment. Thus, the patient was observed without sufficient evaluation of his abdominal blood flow. At the time of recurrence of abdominal symptoms, contrast-enhanced CT showed worsening of stenosis and occlusion in the abdominal aorta and abdominal branch arteries. Therefore, we decided to perform surgical revascularization. Fortunately, the patient recovered without any sequelae.

## Conclusion

Ischemic colitis is a rare complication of Takayasu arteritis. If ischemic colitis is the first symptom of Takayasu arteritis, diagnosis and treatment may be delayed, which may result in death due to intestinal necrosis. Furthermore, there are various causes of abdominal pain in patients with Takayasu arteritis. Thus, it is important to perform various diagnostic tests without delay and establish a definitive diagnosis as soon as possible to improve treatment outcomes.
